# Arrhythmia versus Artefact

**DOI:** 10.1093/ehjcr/ytaf294

**Published:** 2025-07-07

**Authors:** Pujon Purkayastha

**Affiliations:** Cardiology Department, St George’s University, Cranmer Terrace, London SW17 0RE, UK

## Clinical vignette

**Figure 1:**
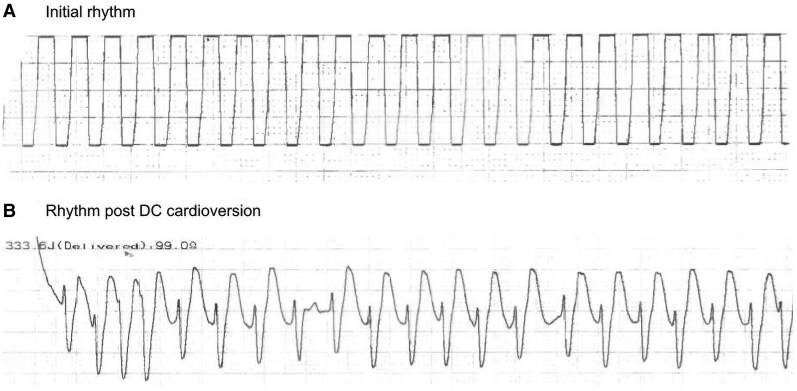
Rhythm strip from external defibrillator.

An 80-year-old male patient presented to the emergency department with abdominal pain. He has a background of ischaemic heart disease (previous coronary artery bypass graft), atrial fibrillation, type 2 diabetes mellitus, and arterial hypertension. Whilst being triaged in the waiting room, he suddenly became grey and clammy. He was rushed to the resuscitation area and placed on a manual defibrillator (antero-lateral pad position), which displayed what appeared to be very organized, symmetrical activity with a sharply defined waveform (see *Figure 1A*). The attending clinicians were uncertain whether this was an accurate reflection of the patient’s cardiac cycle or whether the defibrillator was displaying motion artefact. The patient appeared moribund and had feeble pulses with an unrecordable blood pressure, so the decision was made to manage this as a wide-complex tachycardia with clear adverse features. He was cardioverted at 360 joules (biphasic), and there was an immediate change in the rhythm strip (as shown in *Figure 1B*).

## Question 1

Which of the following best describes the initial ECG rhythm strip:

- Sinus tachycardia with a short PR interval.
- Defibrillator associated artefact.
- Ventricular flutter (VFL).
- Polymorphic ventricular tachycardia.
- Complete AV-dissociation.

Answer: C

### Explanation

*Figure 1A* displays an unusually neat and uniform waveform with an approximate R-R interval of 200 msec. This gives a heart rate of 300 beats per minute. The electrical activity is too organized to be interpreted as artefact and such a misclassification may have disastrous implications for the patient. The most likely cardiac rhythm giving rise to this waveform is ventricular flutter (VFL). Ventricular flutter has previously been attributed to a ventricular tachycardia with an R-R interval of <230 msec.^1^ A noteworthy clinical pearl is that VFL often appears identical when the ECG trace is held upside down.

## Question 2

Which of the following best describes the ECG rhythm strip post DC cardioversion:

- Persistent ventricular tachycardia.
- Sinus tachycardia.
- Pulseless electrical activity.
- Atrial fibrillation with a rapid ventricular response.
- Ventricular escape rhythm.

Answer: D

### Explanation

The rhythm now appears to show a supraventricular tachycardia, which is likely atrial fibrillation in view of the irregular R-R interval and absence of clearly defined *P* waves. The immediate change from a broad to narrow QRS complex tachycardia following DC cardioversion gives credence to the clinical suspicion that the patient was in fact experiencing a ventricular tachyarrhythmia during the initial recording. Options C and E are incorrect as the patient did not completely lose cardiac output.

## Question 3

Which of the following management options is most suitable for this patient:

- Discharge with outpatient follow-up.
- Consider ICD insertion.
- Consider loop recorder implantation.
- Outpatient transthoracic echocardiogram.
- Discharge with antiarrhythmic medication.

Answer: B

### Explanation

The patient in this scenario experienced a haemodynamically unstable ventricular tachyarrhythmia, which if left untreated may have had a fatal outcome. When combined with the patient’s history of extensive ischaemic heart disease, the risks of such an event occuring again is significant. In keeping with the European Society of Cardiology guidelines, the most appropriate management plan in this case would be to consider the insertion of an implantable cardioverter defibrillator (ICD) as part of a secondary prevention strategy.^2^ This patient would also benefit from an inpatient transthoracic echocardiogram and medication optimization prior to discharge.
